# Translating clinicians' beliefs into implementation interventions (TRACII): A protocol for an intervention modeling experiment to change clinicians' intentions to implement evidence-based practice

**DOI:** 10.1186/1748-5908-2-27

**Published:** 2007-08-16

**Authors:** Martin P Eccles, Marie Johnston, Susan Hrisos, Jill Francis, Jeremy Grimshaw, Nick Steen, Eileen F Kaner

**Affiliations:** 1 Centre for Health Services Research, University of Newcastle upon Tyne, 21 Claremont Place Newcastle upon Tyne NE2 4AA, UK; 2 School of Psychology, College of Life Sciences and Medicine, William Guild Building, University of Aberdeen, Aberdeen, AB24 2UB, UK; 3 Health Services Research Unit, Health Sciences Building, University of Aberdeen, Foresterhill, Aberdeen, AB25 2ZD, UK; 4 Clinical Epidemiology Program, Ottawa Health Research Institute, University of Ottawa, 725 Parkdale Ave, Ottawa, ON K1Y 4E9, Canada

## Abstract

**Background:**

Biomedical research constantly produces new findings, but these are not routinely incorporated into health care practice. Currently, a range of interventions to promote the uptake of emerging evidence are available. While their effectiveness has been tested in pragmatic trials, these do not form a basis from which to generalise to routine care settings. Implementation research is the scientific study of methods to promote the uptake of research findings, and hence to reduce inappropriate care. As clinical practice is a form of human behaviour, theories of human behaviour that have proved to be useful in other settings offer a basis for developing a scientific rationale for the choice of interventions.

**Aims:**

The aims of this protocol are 1) to develop interventions to change beliefs that have already been identified as antecedents to antibiotic prescribing for sore throats, and 2) to experimentally evaluate these interventions to identify those that have the largest impact on behavioural intention and behavioural simulation.

**Design:**

The clinical focus for this work will be the management of uncomplicated sore throat in general practice. Symptoms of upper respiratory tract infections are common presenting features in primary care. They are frequently treated with antibiotics, and research evidence is clear that antibiotic treatment offers little or no benefit to otherwise healthy adult patients.

Reducing antibiotic prescribing in the community by the "prudent" use of antibiotics is seen as one way to slow the rise in antibiotic resistance, and appears safe, at least in children. However, our understanding of how to do this is limited.

Participants will be general medical practitioners. Two theory-based interventions will be designed to address the discriminant beliefs in the prescribing of antibiotics for sore throat, using empirically derived resources. The interventions will be evaluated in a 2 × 2 factorial randomised controlled trial delivered in a postal questionnaire survey. Two outcome measures will be assessed: behavioural intention and behavioural simulation.

## Background

### The problem

Clinical and health services research is continually producing new findings that may contribute to effective and efficient patient care. However, despite the considerable resources devoted to this area, a consistent observation is that the transfer of research findings into practice is unpredictable and can be a slow and haphazard process. This phenomenon is apparent across different healthcare settings, countries, and specialties, including the United Kingdom (UK) [1-3], other parts of Europe [4], and the United States of America (USA) [5-7], with obvious implications for patient care. Studies have been unable to explain this variation in terms of either patient or resource factors. Accepting that variation alone does not necessarily represent inappropriate care, a small number of studies have gone on to assess appropriateness [7] and conclude that inappropriate care delivery was occurring.

Symptoms of upper respiratory tract infections (URTIs), are common presenting features in primary care. They are frequently treated with antibiotics, and rates of antibiotic prescribing have been increasing in the UK [8]. However, "the absolute benefits of using antibiotics in the treatment of sore throat are modest. Protecting sore throat sufferers against suppurative and non-suppurative complications in modern Western society can be achieved only by treating with antibiotics many who will derive no benefit." [9]. Reducing antibiotic prescribing in the community by the prudent use of antibiotics is seen as one way to slow the rise in antibiotic resistance [10,11] and appears safe, at least in children [12]. However, our understanding of how best to achieve this is limited [13].

### Implementation research

Implementation research is the scientific study of methods to promote the uptake of research findings, and hence to reduce inappropriate care. It includes the study of influences on healthcare professionals' behaviour, and methods to enable them to use research findings more effectively. Over the last decade, a considerable body of implementation research has been reviewed [14-16]. This research demonstrates that a range of interventions (*e.g*., reminder systems, interactive educational sessions) can be effective in changing health care professionals' behaviour. These studies have substantial heterogeneity within interventions used, targeted behaviours, and study settings that make generalising their findings to routine healthcare settings problematic. This is largely due to the absence of any underlying generalisable framework for both research studies and service settings by which to characterise individuals, settings, and interventions.

The interventions used are usually complex. The framework for phases of investigation of complex interventions suggested by the Medical Research Council (MRC) [17] illustrates the current position with respect to implementation research. Table 1 compares the stages in the evaluation of complex interventions to stages of drug evaluation. To date, most implementation research studies have involved exploratory trials (Phase II) or, more usually, definitive randomized controlled trials (RCTs) (Phase III). While the effectiveness of interventions varies across different clinical problems, contexts, and organizations [18], studies provide scant theoretical or conceptual rationale for their choice of intervention [19]. The current position in the evaluation of implementation strategies is akin to exploring the anti-anginal use of an antihypertensive drug without any understanding of the pharmacodynamics of the drug or the pathophysiology of angina or hypertension, and without Phase I trials of the pharmacodynamics of the drug. Thus, this is an expensive version of trial-and-error, with no *a priori *reason to expect success, nor confidence in replicating success, if achieved.

**Table 1:** Comparison of the stages in an evaluation of complex interventions to stages of drug evaluation.

**Evaluation of drugs**	Pre-clinical	Phase I	Phase II	Phase III	Phase IV
**Evaluation of implementation strategies**	Theory	Modelling	Exploratory trial	Definitive RCT	Long term implementation

To argue against the need for a better theoretical basis for choosing implementation interventions, one would have to suggest that every combination of setting, individual(s), and intervention is unique and must be examined individually – this would require thousands of evaluations and would incur prohibitive costs. The assumption that clinical practice is a form of human behaviour and can be described in terms of general theories relating to human behaviour offers the basis for a generalisable framework. Therefore, factors influencing the effectiveness of interventions could include the beliefs of the healthcare professional, or their perceived ability to control – generalisable concepts that can be used across different interventions, settings, and individuals.

### Using theory to develop implementation interventions: conducting modeling experiments

In order to optimise the number of definitive RCTs studies that will be both costly and time-consuming that need to be conducted, and ensure their generalisability, it is necessary to understand and optimise the 'active ingredients' in professional behaviour change strategies and the characteristics of the settings, targeted professionals, and behaviours that might modify the effectiveness of interventions. Two approaches are necessary to achieve this. One is to develop an understanding of the factors underlying professional behaviour in order to identify what sorts of empirical antecedents should be targeted in implementation interventions (equivalent to the theoretical phase of the MRC Framework, and the subject of our previous work [20]). The other is to develop an understanding of how the elements of the interventions work and can therefore be optimised (the modeling and exploratory trial phases of the MRC Framework).

Almost all of the implementation interventions conducted to date have selected interventions using intuitive/non-theory analytical or empirically successful methods. Three other methods (behavioural change technologies, targeting theoretical antecedents, and targeting empirical antecedents) have been much less developed in implementation research. However, if psychological theory is going to contribute to effective implementation, then targeting empirical antecedents and using behavioural technologies should be the optimum methods of selecting interventions. There are three additional important issues to consider: plausibility and feasibility (both in a development experiment and in service settings), and the method of delivery to maximise efficiency.

### Work leading up to this protocol

#### Using psychological theory to identify salient beliefs that precede the behaviour (empirical antecedents)

We have conducted a number of preliminary studies to investigate the feasibility of using psychological theories in implementation research, and their ability to identify variables that might be targets for interventions. One of these forms the basis of this protocol - a study using the theory of planned behaviour to investigate factors associated with prescribing antibiotics for patients with uncomplicated sore throat by general practitioners (GPs) in Grampian [21]. Literature reviews, non-participant observation, and interviews with GPs were used to develop questionnaires that were distributed to a one in two random sample of GPs in the region, achieving a 70% response rate. Using the theory, we explored the relationships between GPs' perceptions and the strength of their intention to prescribe antibiotics. This allowed us to:

1. Identify whether GPs intended to prescribe antibiotics or not. The majority indicated that they intended to prescribe for less than half of patients presenting with uncomplicated sore throat in the next two weeks.

2. Estimate the overall impact of individual beliefs and perceptions on the strength of their motivation to prescribe; potentially modifiable beliefs accounted for 48% of the variance in GPs' intentions to prescribe.

3. Identify which beliefs had the biggest impact on intention to prescribe antibiotics.

4. Identify discriminant beliefs distinguishing GPs who intended to prescribe from those who did not.

#### A methodology for developing and refining the design of interventions

We have piloted a methodology for developing and refining the design of interventions. In these intervention modeling experiments (IMEs), elements of an intervention are manipulated, within a randomised controlled design, in a manner that simulates a real situation as much as possible; interim endpoints (stated behavioural intention) are measured rather than changes in professional behaviour or healthcare outcome. As such, these studies sit within Modeling and Exploratory Trial Phases of the MRC Framework (Table 1). They offer experimental control and the opportunity to vary elements of an intervention in order to better understand intervening variables and the effect on different outcomes. Compared to large-scale trials, such experiments have potential strengths in terms of their smaller size and shorter timescales.

For the method to be useful, interim endpoints must be predictive of real world outcomes. This is the case for behavioural intention, self-efficacy, and recall and understanding of information. Behavioural intention has been incorporated into virtually all models of health behaviour as the single best predictor of subsequent health behaviour [22]. The predictive ability of intention has been demonstrated by reviews of both observational [23-25] and experimental studies [26], with intention explaining 20% to 40% of variance in behaviour. Self-efficacy has also been widely incorporated into models predicting behaviour because of its reliable predictive effect [27]. In interventions providing information, recall of that information has been shown to be important to achieve behaviour change [28].

We have undertaken two pilot studies that demonstrate the feasibility of the method [29,30]. In the first, we designed an intervention to reduce the frequency of extraction of third molar teeth by selecting the behaviour change technique "generating alternative behaviours" [29]. General dental practitioners (GDPs) were randomly selected from the Scottish Dental Practice Board Register and allocated to control or intervention groups, the latter receiving a postal behavioural manipulation, and both groups responding to a postal questionnaire. Subjects in the intervention group were asked to generate a list of management alternatives to third molar extraction prior to being asked to record their third molar extraction intention, while subjects in the control group were not. The intervention group had statistically significantly less intention to extract third molars than the control group, despite similar knowledge of management alternatives. In the second, we simulated an empirically successful intervention [30]. In this study, we investigated the effectiveness of audit and feedback and educational reminder messages in changing simulated x-ray test ordering by GPs. Baseline rates of x-ray test ordering were established in a postal survey based upon GPs' intentions to request x-rays based upon patient vignettes. GPs were then sent simulated results of any x-rays that they had requested. In addition, they were randomised (within a 2 × 2 factorial design) to receive or not 'audit & feedback' (comparative group feedback generated from the first round responses) or 'educational messages' on their x-ray result forms. Both interventions were effective in changing behavioural intentions.

This preliminary work forms the basis of the present protocol, the purpose of which is to use psychological theory in the design and experimental evaluation of behavioural interventions to change professional practice.

### Aims of this protocol

The aims are 1) To develop interventions to change beliefs that have already been identified as antecedents to antibiotic prescribing for sore throats, and 2) to experimentally evaluate these interventions to identify those which have the largest impact on behavioural intention and behavioural simulation.

## Methods

### Clinical activity, setting, and participants

We will use the management of uncomplicated sore throat in general practice as the clinical focus for this work. Participants will be general medical practitioners.

### Design

Two interventions will be developed to address the discriminant beliefs in the prescribing of antibiotics for sore throat. Appropriate intervention components will be selected from a number of available evidence-based behavioural technologies. The design of the interventions will incorporate these techniques and will be further informed by the empirical findings of our previous studies. The interventions will be evaluated in a 2 × 2 factorial randomised controlled trial delivered in a postal questionnaire survey.

### Interventions

Our previous work [21] has identified eight discriminant beliefs that distinguish between GPs who do (intenders) and do not intend (non-intenders) to prescribe antibiotics for patients with uncomplicated sore throat (Table 2). We will make the assumption that altering these beliefs will change intentions to manage URTI without prescribing antibiotics, and we will therefore design the interventions to change these beliefs. Therefore, it will be possible to test the assumption empirically by applying a mediational analysis to explain intervention effects. Two theory-based interventions that incorporate behaviour change technologies will be designed to promote the management of URTI presenting in primary care without prescribing antibiotics.

**Table 2:** Discriminant beliefs that distinguish between GPs who do (intenders) and do not intend (non-intenders) to prescribe antibiotics for patients with uncomplicated sore throat.

**Behavioural beliefs**
Prescribing an antibiotic for these patients will reduce their risk of developing minor complications such as otitis media and sinusitis
Prescribing an antibiotic for these patients is cost efficient
Prescribing an antibiotic for these patients will reduce the time taken for their sore throat to resolve
**Outcome evaluation**
The problems of antibiotic resistance for these patients does not concern me greatly
**Control beliefs**
If a patient asks for an antibiotic, then I will prescribe one whether it is medically indicated or not
I am more inclined to prescribe an antibiotic for patients of a lower social class
Because I don't know the cause of these patients' sore throats, I will prescribe an antibiotic so that I don't miss something
In most cases, the patient will finish the course of antibiotics I prescribe

### Outcome measurement

Two outcome measures will be assessed, behavioural intention and behavioural simulation. We will measure behavioural intention using the standard methods used in investigations based on the theory of planned behaviour using rating scales of likelihood, frequency, or agreement with statements or questions about intention (*e.g*. Out of the next 10 patients you see with acute sore throat, how many do you intend to prescribe antibiotics for? Score 0 – 10). To measure behavioural simulation, participants will be asked to respond to written scenarios describing patients presenting with sore throat in general practice. The scenarios will reflect the range of patients and clinical features that present in general practice informed by qualitative work conducted in our previous work [21]. Participants will be asked to write on a simulated set of notes the relevant management they would use.

### Process measurement

We will examine whether the interventions affect the discriminant antecedents identified in the previous theory of planned behaviour study (Table 2). We have piloted these methods successfully [29,31]. The results will be explored using the Baron and Kenny methodology for mediational analyses [32], which incorporates the Sobel test, to ascertain the extent to which these antecedent beliefs mediated effects on outcomes within these experiments. Where possible, the measurement will be made twice, with these process measures assessed both before and following the intervention. The time between measurements will be six to eight weeks.

### Delivering the modeling experiment

The experimental materials will be delivered by post. The experiment will be embedded within a questionnaire survey which will be administered twice, once before the intervention and once immediately following the intervention. Based on our previous experience, we plan that subjects will receive a letter of invitation, a set of instructions, and individually packaged set of materials for measuring behavioural simulation and intention that they will be asked to read in this order. On the second administration of the survey, they will also receive the intervention which they will be asked to complete prior to completing the process and outcome measures. Two reminders will be mailed to non-responding clinicians. Given our experience of the response rate in our previous study [31], we plan to offer a £10 incentive to each subject to increase response rates [33,34].

### Sample size and analysis

In a 'definitive trial', there is inherent variability in the number of patients who consult with each condition, and the characteristics of these patients vary from doctor to doctor and from year to year. By giving all subjects in the experiment the same context in which to examine behavioural intention, we have eliminated these two sources of variation. Therefore, if we use the same outcome in both the trial and in the IME, we would expect its standard deviation to be smaller in the IME than in the trial. Thus, a given shift in outcome (difference between two groups) represents a much larger effect size (difference in outcome divided by the standard deviation) in the IME than in the trial. Thus, if a trial were to produce a moderate effect size we might expect a large effect size in the IME. The IME will be powered to detect difference between each of the active intervention groups and the control group. Using standard methods for a continuous outcome, we need 50 subjects per group to have 80% power of detecting an effect size of 0.8 using a significance level of 2.5%, giving a total sample size of 200 for the experiment. We will over-sample, using an initial sample of 800, to ensure that we achieve this final sample size. This will be adjusted in the light of the impact of the incentive. Groups will be compared using methods appropriate for comparing independent samples (t-tests to compare two groups, analysis of covariance to compare groups adjusting for differences in baseline performance).

### Ethics approval

The study has ethical approval from the Northern and Yorkshire Multi-Centre Research Ethics committee. (REC Reference: 05/MRE03/11).

## Competing interests

The author(s) declare that they have no competing interests.

## Authors' contributions

All authors contributed to the conception and design of the study and approved the submitted draft.
